# Specific imaging features indicate the clinical features of patients with hepatic perivascular epithelioid cell tumor by comparative analysis of CT and ultrasound imaging

**DOI:** 10.3389/fonc.2022.908189

**Published:** 2022-10-17

**Authors:** Xudong Gao, Hewen Tang, Jianying Wang, Qian Yao, Hong Wang, Yan Wang, Mingming Ma, Wei Yang, Kun Yan, Wei Wu

**Affiliations:** ^1^ Department of Ultrasound, Key Laboratory of Carcinogenesis and Translational Research (Ministry of Education), Peking University Cancer Hospital and Institute, Beijing, China; ^2^ Hepatology Department and Emergency Department, The 5th Medical Center of PLA General Hospital, Beijing, China; ^3^ Department of Information, Medical Supplies Center of PLA General Hospital, Beijing, China; ^4^ 4Department of Pathology, Key Laboratory of Carcinogenesis and Translational Research (Ministry of Education), Peking University Cancer Hospital and Institute, Beijing, China

**Keywords:** perivascular epithelioid cell tumor (PEComa), contrast-enhanced computed tomography (CECT), contrast-enhanced ultrasound (CEUS), focal liver lesion (FLL), rare liver tumors

## Abstract

**Objective:**

The objective of the study was to explore the CT and ultrasound features and clinical significance of perivascular epithelioid cell tumor (PEComa) of the liver.

**Methods:**

Eleven hepatic PEComa patients treated in our hospital were retrospectively analyzed based on the characteristics of the imaging results of the patients, including conventional ultrasound, CDFI, contrast-enhanced ultrasound (CEUS), and contrast-enhanced CT (CECT).

**Results:**

CT scans showed that all lesions were hypodense. Ultrasonography showed that lesions were either hyperechoic (4/11, 36.36%), hypoechoic (4/11, 36.36%), isoechoic (1/11, 9.09%), or heterogeneously echoic (2/11, 18.18%). CDFI showed that most of the lesions had an abundant blood supply (9/11, 81.82%). Whether on CT scan or ultrasonography, the margins of the lesions were dominated by clear margins. Ultrasonography revealed more features: hyperechoic patterns around lesions (3/11, 27.27%) and lateral shadow (5/11, 45.45%). The CDFI showed that large blood vessels were observed around the lesions (9/11, 81.82%). CECT shows two enhancement patterns: “fast in and fast out (FIFO)” (8/11, 72.72%) and “fast in and slow out (FISO)” (3/11, 27.27%). CEUS shows that all lesions had the enhancement pattern of “FISO,” which was different from CECT. All lesions displayed rapid enhancement during HAP in CEUS during 7–20 s. Four patients (36.36%) washed out at 60–180 s, another four (36.36%) washed out at 180–300 s, and the remaining three patients (27.27%) showed no signs of washout even at 360 s.

**Conclusion:**

Some imaging features, such as clear margins, peripheral hyperechoic around the lesion, lateral shadow, the large blood vessels around lesions, and the “FISO” enhancement pattern, may indicate expansive growth of the tumor and be helpful in the diagnosis of PEComa. Ultrasound images may provide more details for clinical reference.

## Introduction

PEComa (perivascular epithelioid cell tumor) is a mesenchymal tumor with histological and immunophenotypic features of perivascular epithelioid cells (1). In 1992, Bonetti et al. described this tumor as originating from the liver for the first time (2). Four years later, Zamboni introduced the name PEComa (3). The pathological features are often characterized by abundant cytoplasm and clear eosinophilic granules, and they show focal association with blood vessel walls and usually express melanocytic markers (HMB-45 and Melan.A) and smooth muscle markers (SMA) (1). PEComa includes angiomyolipoma (AML), clear cell/sugar tumor of the lung, lymphangioleiomyomatosis, clear cell myomelanocytic tumor, and other histologically and immunohistochemically similar tumors (1). The tumor mostly occurs in the uterus but also in other organs such as the kidneys, bladder, prostate, lungs, pancreas, liver, etc. (4). It is more common in young women, with a male-to-female ratio of 1:6 (5).

PEComa tumors originating from the liver are rare, which are often misdiagnosed because of the atypical imaging features (6). As we all know, the main clinical diagnostic approaches for liver lesions are based on imaging examinations (7). For example, the clinical diagnosis of hepatocellular carcinoma (HCC) is mainly based on the history of liver disease of the patient and the imaging characteristics of contrast-enhanced imaging examinations, on which the lesions share an imaging enhancement pattern of “fast in and fast out” (7). A few studies have explored some of the imaging features of PEComa and have found some atypical imaging features. In contrast-enhanced computed tomography (CECT) studies, the arterial phase showed intensive saturation of the vessels in the periphery of the tumor, and the portal and delayed phases showed a marked decrease in the amount of contrast (8, 9). In MR studies in the T1 phase, a significant intensity was visible, while the T2 intensity decreased (9, 10). However, the imaging characteristics of PEComas are unclear, especially those related to contrast-enhanced ultrasound (CEUS).

Therefore, further research on the imaging characteristics of PEComas as a rare disease is of significant clinical value, which is beneficial to our in-depth understanding of the occurrence and development of this disease and the diagnosis, treatment, and prognosis of this disease in clinical work. In our study, 11 patients with hepatic PEComa treated in our hospital over the past ten years were retrospectively analyzed based on the characteristics of their imaging results, including conventional ultrasound (US), color Doppler flow imaging (CDFI), CEUS, and CECT, and pathologic analysis. Some results may provide important references for our clinical work.

## Materials and methods

### Inclusion and exclusion criteria

The data of all patients with PEComa admitted to our hospital from January 2008 to December 2018 were collected. To be included in the study, the patients had to meet the following inclusion criteria: (1) the patient had undergone a liver CEUS examination; (2) the patient had received an abdominal CECT at the same time as the CEUS examination; and (3) the lesion was surgically removed, and the diagnosis of PEComa was confirmed by pathology. The patient was excluded if one of the following exclusion criteria was met: (1) the patient did not receive a CEUS and a concurrent abdominal CECT; or (2) the pathologic diagnosis was not definitive.

### Abdominal CECT scan

A Lightspeed VCT 64-slice CT scanner (GE Healthcare, Boston, US) was used. The scan range was from the top of the liver to a section 2 cm lower than the inferior border of the liver. A contrast agent containing 1%–2% iodine was orally administered before the examination. The slice thickness was 5 mm without reconstruction intervals. A plain CT scan was performed first, and then a bolus injection of 80–100 ml of iopromide (a contrast agent containing 300 mg/ml of iodine) was administered *via* the cubital vein using a high-pressure syringe at 3.5 ml/s. Triphasic, dynamic CT was performed. The hepatic arterial phase (HAP), portal venous phase (PVP), and delayed phase were 20–25 s, 60 s, and 90–120 s, respectively, after the start of the injection.

### Conventional ultrasound and CEUS

The GE (GE Healthcare, Milwaukee, Wisconsin, USA) LOGIQ E9 and LOGIQ 9 color Doppler ultrasound scanners were used in this study. The scanner is equipped with C1-5 (LOGIQ E9) and 4C (LOGIQ 9) abdominal probes. The frequencies were 2.8–5.0 MHz and 2.0–4.0 MHz for LOGIQ E9 and LOGIQ 9, respectively. Conventional ultrasound, CDFI, and CEUS were performed to examine the livers of the patients. SonoVue (Bracco SpA, Milan, Italy) was used as the contrast agent and configured according to the instructions. The patient received a bolus injection of SonoVue through the anterior cubital vein, with 1.5–2.4 ml given at each injection. The lesions were inspected continuously throughout the HAP (0–30 s) and PVP (31–120 s). A full liver scan was performed at the delayed phase (120–360 s) to detect whether there were other abnormal lesions with hyper-enhancement or hypo-enhancement. Ten minutes after the first CEUS scan, a second CEUS scan was performed on the suspected lesion to assess the status of enhancement and washout. Each CEUS scan lasted for at least 6 min, and the footage was stored. The imaging characteristics of the lesions examined using conventional ultrasound, CDFI, and CEUS were recorded.

### Image analysis

All images were independently reviewed by two radiologists or sonographers who had 10–20 years of experience in the interpretation of abdominal CT/CECT and ultrasound/CDFI/CEUS images. The final decisions were reached by consensus.

### Pathological examination

Tumors surgically removed from all patients were subjected to pathologic examinations. Histopathological evaluation included routine hematoxylin and eosin (HE) staining and immunohistochemical staining. Melanocytic markers include HMB-45 and Melan.A and smooth muscle markers include SMA. Pathological examinations and specimen handling were performed by experienced liver pathologists. The cells were positive for human melanoma black-45 (HMB45) and Melan.A on immunohistochemical staining, confirming that the lesion was a PEComa.

### Ethics approval

This study received institutional review board approval and informed patient consent.

### The statistical methods

SPSS 13.0 software was used for data analysis. Continuous variables were expressed as means ± SD. The comparison between the two examination methods was performed by McNemar’s test. P <0.05 was statistically significant.

## Results

### Clinical data of the patients

A total of 11 patients were included in this study. All patients were diagnosed with PEComa by pathological examination. The clinical characteristics of the patients are shown in **Table 1**. There were eight females and three males, with an average age of 46.73 ± 13.33 years old (range from 27 to 67 years old). None of the patients had hepatotropic viral infections or liver cirrhosis. Two patients had histories of hypertension, and the other two had diabetes. Clinical symptoms were nonspecific and included abdominal distension, abdominal pain, or no apparent clinical symptoms. Nine patients (81.81%) had a single liver lesion, whereas two patients (18.18%) had multiple lesions. The diameter of the lesions was 58.91 ± 32.36 mm using an ultrasonic technique. The serum levels of the tumor markers alpha-fetoprotein (AFP), carcinoembryonic antigen (CEA), carbohydrate antigen 19-9 (CA19-9), and cancer antigen 125 (CA125) were all within the normal range.

**Table 1:** Summary of general information and clinical features of patients.

Clinical characteristics	value
1. Age (means ± SD, years)	46.73 ± 13.33
2. Gender: Male/female (%)	3/8 (27.27%/72.73%)
3. Combined with hepatophilic virus infection (HBV/HCV)	0/0
4. Cirrhosis (no/yes)	11/0
5. Past medical history: Hypertension/diabetes/no other history (%)	2/2/8 (18.18%/18.18%/72.73%)
6. Lesion size* (means ± SD, mm)	58.91 ± 32.36 mm
7. Number of lesions: single/multiple* (%)	9/2 (81.82%/18.18%)
8. Tumor markers (AFP/CA199/CEA/CA125)	Normal

*There were two patients, each of whom had two lesions, and the size of the lesions was calculated as the sum of the largest diameters of the two lesions.

### In the evaluation of lesion, non-enhanced imaging showed significant differences, and CDFI may reveal more useful information

**Figure 1:**
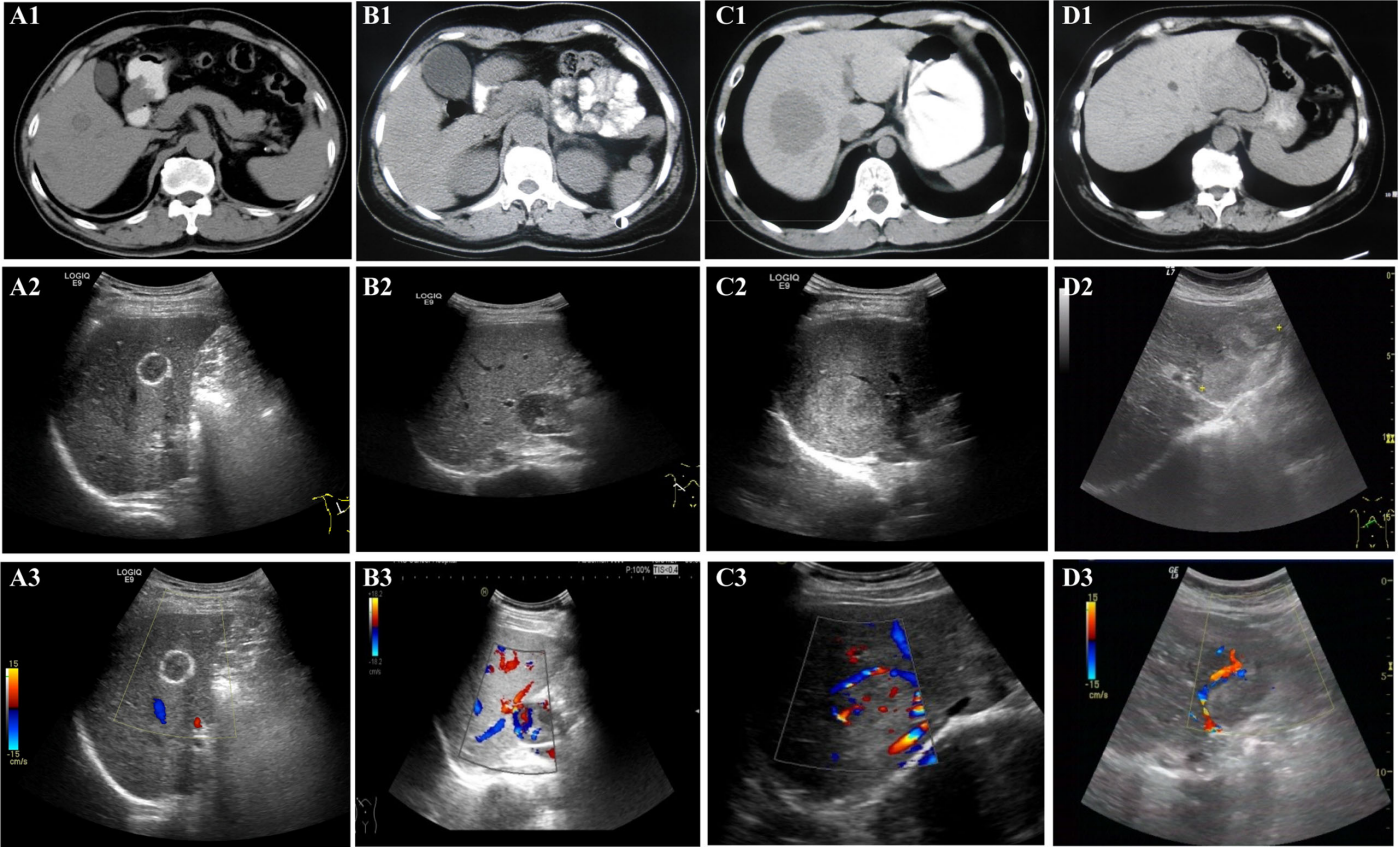
Non-enhanced imaging (CT, US, and CDFI) appearance. All patients underwent non-enhanced imaging examination, including CT, US, and CDFI. A1 (CT), A2 (US), and A3 (CDFI) are images of lesions in the patient 10. B1 (CT), B2 (US), and B3 (CDFI) are images of lesions in the patient 2. C1 (CT), C2 (US), and C3 (CDFI) are images of lesions in the patient 1. D1 (CT), D2 (US), and D3 (CDFI) are images of lesions in patient 4. For CT, **(A1, B1, C1,** and **D1)**: CT scans showed that all lesions were hypodense. For US, **(A2)**: US showed the lesion with hyper-echoic ring around and iso-echo inside, lateral shadow, clear boundary and regular shape. **(B2)**: US showed hypoechoic lesions with clear boundary and regular shape; **(C2)**: US showed hyperechoic lesion with clear boundary and regular shape; **(D2)**: US showed that the lesion were heterogeneously hyper-echoic, unclear boundary and irregular shape. For CDFI, **(A3)**: CDFI showed no obvious blood flow signal inside and around the lesion; **(B3, C3, D3)**: CDFI showed that there were large blood vessels and blood flow signals inside the lesion.

In the evaluation of lesions, CT, US, and CDFI were performed. These results are shown in **Tables 2**, **3**. First, in the detection of lesion size, the diameter of the lesion detected by CT was 52.45 ± 30.26 mm; however, the diameter detected by US was 58.91 ± 32.36 mm. The two results were significantly different. Second, CT scans showed that all lesions were hypodense, showing good consistency (**Figure 1**). However, the results for the US were markedly different (**Figure 1**). These lesions did not show apparent consistency, and their image performance included hyperechoic, hypoechoic, isoechoic, and heterogeneously echoic. Four patients (4/11, 36.36%) were hyperechoic; one patient (1/11, 9.09%) was isoechoic; four patients (4/11, 36.36%) were hypoechoic; and two patients (2/11, 18.18%) were heterogeneously echoic. Importantly, CDFI showed that most of the lesions had an abundant blood supply (9/11, 81.82%), but the blood supply appeared insufficient in two patients (2/11, 18.18%). This feature may help patients in the selection of treatment methods.

**Table 2:** Summary of CT/CECT findings in patients.

Case	Lesion size (cm)	Margin	Plain CT	HAP	PVP	Delayed phase	Type
1	5.2 × 4.4	clear	low-density	heterogeneous enhancement	heterogeneous enhancement	high-density	FISO
2	4.0 × 2.7	blurred	low-density	heterogeneous enhancement	low-density	low-density	FIFO
3	4.5 × 4.3	clear	low-density	homogeneous enhancement	low-density	low-density	FIFO
4	5.6 × 6.3	clear	low-density	heterogeneous enhancement	iso-density	iso-density	FISO
5	6.4 × 6.1	clear	low-density	heterogeneous enhancement	high-density	high-density	FISO
6	4.5 × 4.8	clear	low-density	heterogeneous enhancement	low-density	low-density	FIFO
7	5.3 × 4.0	blurred	low-density	heterogeneous enhancement	low-density	low-density	FIFO
8	3.1 × 2.2	clear	low-density	heterogeneous enhancement	low-density	low-density	FIFO
9	14.1 × 10.0	clear	low-density	heterogeneous enhancement	low-density	low-density	FIFO
10	1.9 × 1.7	clear	low-density	homogeneous enhancement	low-density	low-density	FIFO
11	3.2 × 2.5	blurred	low-density	significant enhancement with a visible patchy area of slightly low density in the center	low-density	low-density	FIFO

FIFO, “fast in and fast out”; FISO, “fast in and slow out”; HAP, hepatic arterial phase; PVP, portal venous phase.

**Table 3:** Summary of Ultrasound/CDFI/CEUS findings in patients.

Case	Lesion size (cm)	Echo	Margin	CDFI	Halo sign	Large blood vessel*	Enhancement Pattern	Wash-in (s)	Wash-out (s)
1	5.0 × 4.5	hyperechoic	blurred	+	No	Yes	homogeneous enhancement	8–13	no^b^
2	4.2 × 3.7	hypoechoic	clear	+	No	Yes	homogeneous enhancement	15–20	68
3	4.6 × 4.1	hypoechoic	clear	+	No	Yes	homogeneous enhancement	14–20	60
4	6.8 × 5.5	hyperechoic	clear	+	No	Yes	homogeneous enhancement	15–20	300
5	7.6 × 5.0	heterogeneously echoic	blurred	+	No	Yes	homogeneous enhancement	10–16	190
6	5.3 × 5.3	heterogeneously echoic	clear	+	No	Yes	homogeneous enhancement	10–14	200
7	6.7 × 4.2	hypoechoic; hyperechoic patterns around the lesions	blurred	+	No	Yes	homogeneous enhancement	10–15	no^b^
8	3.6 × 2.8	hypoechoic	blurred	+	No	Yes	homogeneous enhancement	11–15	300
9	15.0 × 7.7	hyperechoic	clear	+	No	Yes	homogeneous enhancement	8–12	no^b^
10	2.7 × 1.8	hyperechoic; hyperechoic patterns around the lesions	clear	−	No	No^a^	homogeneous enhancement	10–15	84
11	3.3 × 2.6	isoechoic; hyperechoic patterns around the lesions	clear	−	No	No^a^	homogeneous enhancement	7–13	73

*Larger blood vessels around lesions; Yes, larger blood vessels were observed around lesions; No^a^: no blood vessels were observed around lesions; ±, abundant blood supply/insufficient blood supply; no^b^, no signs of washout even at 360 s.

### In the evaluation of the margin of the lesions, non-enhanced imaging often showed associated features of expansile growth, and ultrasound revealed more detail

In the evaluation of the margin of the lesions, the two examination methods showed some features that suggested the possibility of expansile growth (**Tables 2**, **3**). Whether on CT scan or ultrasonography (**Figure 1**), the margins of the lesions were dominated by clear margins:In eight patients, the margins of the lesionswere clear (8/11, 72.73%) (in the other three patients, the margins of the lesions were blurred (3/11, 27.27%)); and US showed that the lesions in seven patients (7/11, 63.64%) had clear margins (in the other four patients, the margins of the lesions were blurred (4/11, 36.36%)). But the results of the two imaging examinations were not completely consistent. At the same time, the US revealed more features (**Figure 1**). Some lesions showed hyperechoic patterns around themselves (3/11, 27.27%). Almost half of patients (5/11, 45.45%) had lesions with lateral shadow. None of the lesions displayed any obvious halo signs, which is characteristic of infiltrative growth of malignant tumors, including hepatocellular carcinoma. In addition, some larger blood vessels were observed around the lesions, which is another characteristic of these lesions (**Figure 1**). In the CT scan, there were four patients with this characteristic. However, US, especially CDFI, showed that large blood vessels with diameters greater than 2 mm were observed around the lesions in nine patients (9/11, 81.82%). Only two patients were absent (2/11, 18.18%). Finally, although some lesions were larger, both imaging studies showed no enlarged lymph nodes and no distant metastases. The above features may indicate that the tumor may show expansive growth. and US images may provide more details for clinical reference.

### Contrast-enhanced CT shows two enhancement patterns

**Figure 2:**
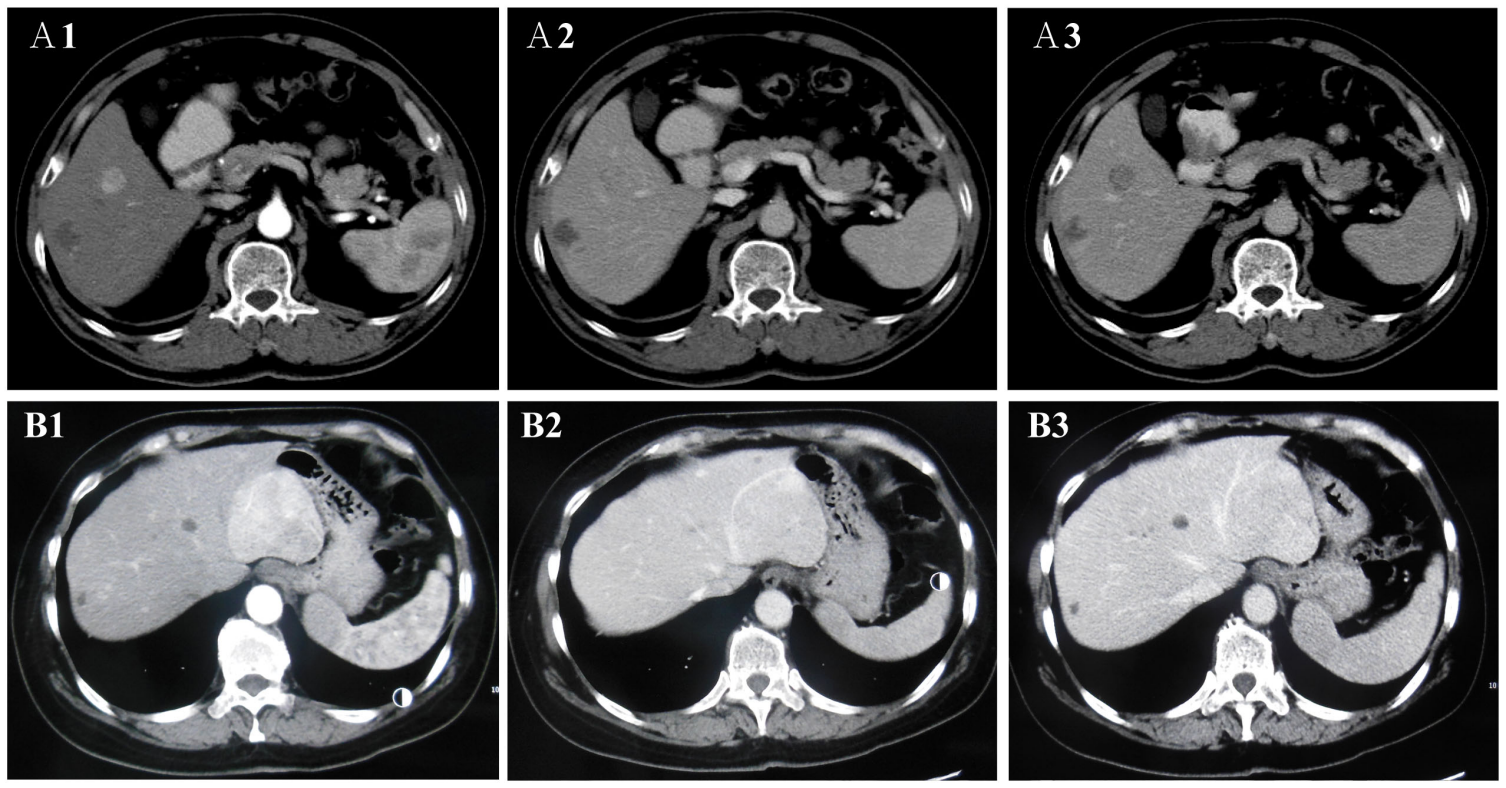
The two enhancement patterns of CECT. The enhancement patterns of CECT included two types: “fast in and fast out” (FIFO) and “fast in and slow out” (FISO). A1, A2, and A3 are CECT images of lesions in the patient 10. **(A1)** In arterial phase, the lesions were obviously enhanced with high-density; **(A2, A3)** In portal vein phase and delayed phase, the lesion was observed low-density. CECT showed “FIFO” enhancement pattern. B1, B2, and B3 are CECT images of lesions in the patient 4. **(B1)** In arterial phase, the lesions were obviously enhanced with high-density and large blood vessels were observed at the edge of the lesion; **(B2)** In portal vein phase and delayed phase, the lesion was observed iso-density and low-density. **(B3)** In delayed phase, the lesion was observed low-density. CECT showed “FISO” enhancement pattern.

All patients received an abdominal CECT scan (**Table 2** and **Figure 2**). First, all lesions showed obvious enhancement during the HAP. Eight (8/11, 72.73%) of the patients displayed heterogeneous enhancement; two patients (2/11, 18.18%) showed homogeneous enhancement; and one patient (1/11, 9.09%) showed significant enhancement with a visible patchy area of slightly low-density in the center. Second, during the PVP and delayed phase, low-density lesions were observed in eight patients (8/11, 72.73%), high-density lesions were observed in two patients (2/11, 18.18%), and iso-density was observed in one patient (1/11, 9.09%). Therefore, the enhancement pattern was divided into two types: “fast in and fast out” (FIFO) and “fast in and slow out” (FISO) (**Figure 2**). For all patients with hepatic PEComa that we investigated, the former pattern was found in eight patients (8/11, 72.72%), while the latter was only observed in three patients (3/11, 27.27%). All lesions with blurred margins showed the enhancement pattern of “fast in and fast out.” CECT identified large blood vessels around the lesions in four patients.

### CEUS shows the enhancement pattern of “fast in and slow out,” which was different from CECT

**Figure 3:**
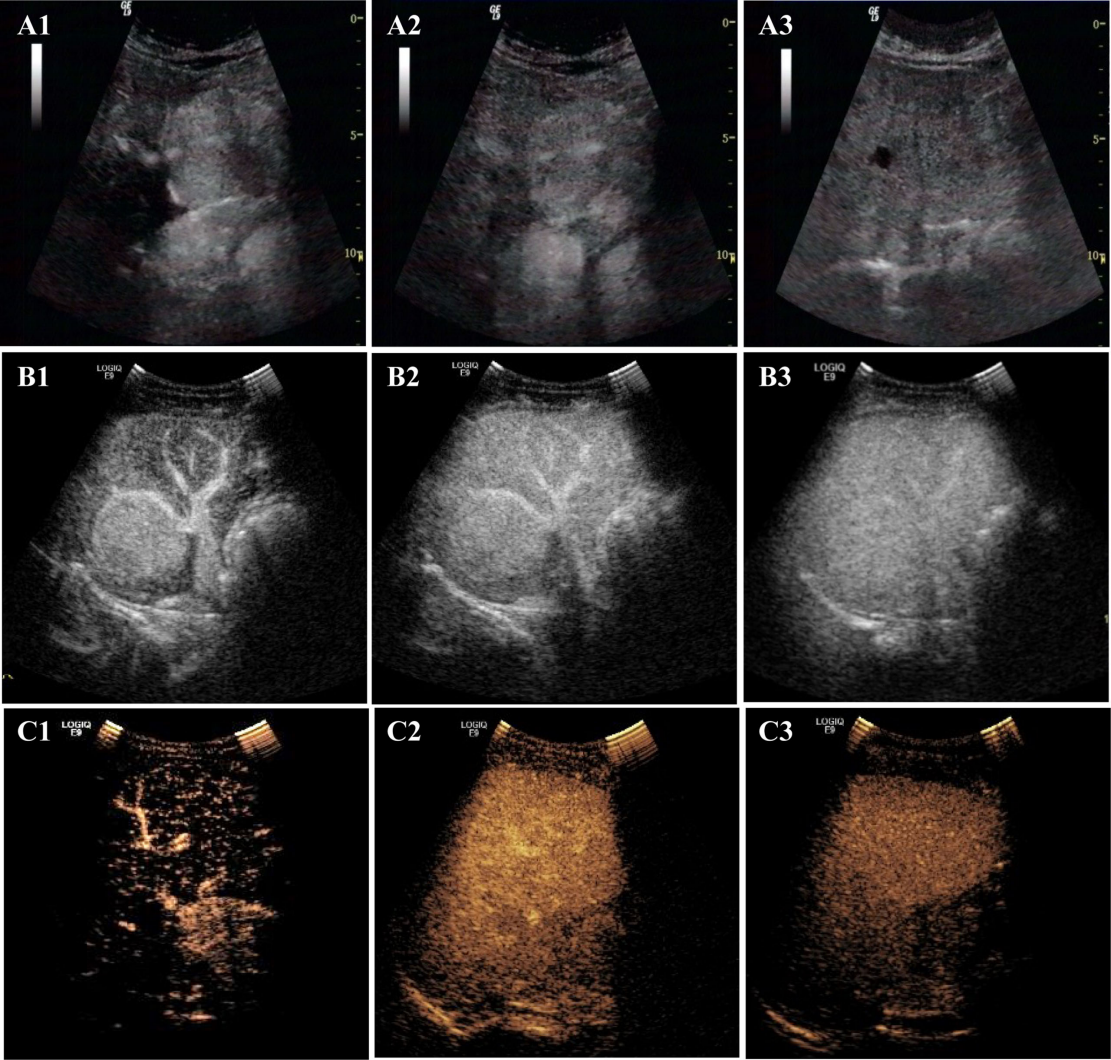
The enhancement patterns of CEUS. The enhancement pattern of CEUS was “fast in and slow out” (FISO). A1, A2, and A3 are CEUS images of lesions in the patient 4: **(A1)** rapid enhancement in the arterial phase; **(A2)** slightly higher enhancement in the portal phase; **(A3)** the lesion began to wash out at 300s in delayed phase. B1, B2, and B3 are CEUS images of lesions in the patient 1. **(B1)** rapid enhancement in arterial phase with large blood vessels; **(B2)** in portal phase, the lesion was still hyper-enhanced; **(B3)** the lesion did not wash out at 360s in delayed phase. C1, C2, and C3 are CEUS images of lesions in the patient 2. **(C1)** rapid enhancement in arterial phase; **(C2)** the lesion began to wash out at 68 s in portal phase fade; **(C3)** The lesion was observed with low-enhancement in delayed phase.

After US, each patient also underwent a CEUS examination (**Table 3** and **Figure 3**). CEUS can display the entire dynamic process of contrast enhancement and washout in the lesions. First, all the lesions displayed rapid and homogeneous mass-like enhancement during the HAP on CEUS, which was called “fast in” and significantly different from CECT (*p* = 0.004) (**Table 4**). The time of enhancement was approximately 7–20 s, initiating at approximately 7–15 s and reaching whole-lesion enhancement after 4–6 s. Second, the washout started late, and in some patients, it never occurred. These were called “slow outs.” Among all patients, no washout was observed in all lesions within the first 60 s; in four patients, the washout started at 60–180 s (36.36%), and in another four (36.36%); it started at 180–300 s. The remaining three patients showed no signs of washout even at 360 s (27.27%) (**Table 3**). Therefore, on CEUS, all lesions showed an enhancement pattern of “fast in and slow out” (FISO), which was consistent with the pathological features reported in the literature (11); that is, these imaging features were more suggestive of expansile growth and benign lesions (12, 13). These results were significantly different from CECT (*p* = 0.008) (**Table 4**).

**Table 4:** Comparative analysis of CT/CECT and US/CDFI/CEUS.

characteristics	CT/CECT	US/CDFI/CEUS	Consistent	Inconsistent	*P*-value
Clear (CT/US)	8	7	5	5	0.654
Blurred (CT/US)	3	4	1		
homogeneous enhancement (CECT/CEUS)	2	11	2	9	0.004
heterogeneous enhancement (CECT/CEUS)	9	0	0		
FIFO (CECT/CEUS)	8	0	0	8	0.008
FISO (CECT/CEUS)	3	11	3		
Large blood vessel around lesions	4	9	4	5	0.062
No large blood vessel around lesions	7	2	2		

Consistent: the results of the two examination methods were consistent. Inconsistent: the results of the two examination methods were inconsistent.

## Discussion

Focal lesions are common among liver diseases, and the most common are HCC, hepatic hemangioma, and hepatic cysts. All these diseases present some apparent features on imaging examination, which is very helpful for noninvasive clinical diagnosis of focal liver lesions (7). However, PEComas, as a rare focal lesion of the liver, is difficult to diagnose based on clinical data. The reason is that their imaging characteristics have not been identified, possibly due to the rarity of cases. The diagnosis of this disease relies mainly on pathology. To better understand the imaging features of hepatic PEComas and provide evidence to support an early and conclusive diagnosis to reduce the risks of invasive test procedures, we collected the imaging data of patients with hepatic PEComas treated in our hospital during the past ten years and performed a comparative analysis of the imaging features of different imaging exams. This study may provide some helpful information for the clinical diagnosis and treatment of hepatic PEComas.

The non-enhanced scan is a commonly used screening method in clinical practice (7). In this study, we applied two non-enhanced scans, CT and US/CDFI, and performed a comparative analysis. The two images simultaneously indicate 1) most of the lesions had clear margins; 2) larger blood vessels were observed around larger lesions (US was significantly better than CT for this feature). Unlike the CT, the US also showed some special features. First, in some patients, the echoes surrounding the lesions appeared to be significantly hyperechoic, even though the lesion itself was only hypoechoic, isoechoic, or slightly hyperechoic. This phenomenon may indicate a potential feature of PEComa. Second, the lateral shadow of lesions was observed in some patients. In addition, all lesions had no obvious halo signs, which is a common imaging manifestation of malignant tumors (14). The above characteristics suggest that the biological behavior of the tumor is the possibility of expansile growth. Expansile growth is the main growth pattern of benign tumors. This feature was consistent with the pathological features of hepatic PEComas reported in the literature (15), which led us to believed that hepatic PEComas is a potentially malignant tumor with a good clinical prognosis (16). This feature may suggest relevant genomic changes. The further study of these changes may have important implications for understanding tumor progression (17–19).

In the examination of the lesions, all lesions showed low density, which was an important feature of PEComa by CT scan. This was similar to the literature report (20). We did not find any obvious cystic lesions, which is a slightly different finding from what has been reported in related literature (21, 22). By conventional US, the lesions showed various echogencities (such as hypoechoic, isoechoic, and slightly hyperechoic) with no apparent specificity.

The blood supply of the lesion is closely related to the growth of the lesion. CDFI provides a good technical means for blood supply tests. For PEComas, CDFI demonstrated that lesions had an abundant blood supply, similar to primary HCC. The feature of abundant blood supply may be helpful for the choice of treatment methods, such as anti-angiogenic therapy (17, 23, 24) or vascular interventional therapy. However, further research is needed to prove it. CDFI showed that the larger blood vessels around the lesion may be another potential feature of PEComa. Hassania made similar findings through a case report of PEComa (25). However, few HCCs are surrounded by large blood vessels. This may be a special feature of PEComas. Unfortunately, in previous examinations, the features of the large blood vessels surrounding the lesion were not investigated, and whether these vessels were related to tumor growth is not known. Considering that PEComa is a vascular epithelial tumor, the lesion may be caused by pathological changes in the peripheral epithelial cells of large blood vessels. Further studies are needed to provide a clear answer to this question.

In clinical practice, enhanced imaging examinations have become one of the most important test approaches for detecting and diagnosing focal liver lesions (7). A large number of HCC and hepatic hemangioma rely on abdominal CECT or contrast-enhanced MRI examinations for diagnostic confirmation. These lesions also show specific characteristics. For example, patients with HCC often have a “fast in and fast out” pattern of lesion enhancement (7). These imaging tests have not only greatly improved the rate of disease detection and the accuracy in identifying lesions, but also reduced patient pain and suffering associated with invasive tests. However, contrast-enhanced imaging examinations of PEComa patients have not identified any obvious features of the lesions, which increase the challenge in the clinical diagnosis of PEComa. Therefore, further understanding of the imaging characteristics of PEComa on contrast-enhanced imaging exams is of great value for clinical treatment of the disease.

In this study, we retrospectively analyzed the imaging data of all PEComa patients. CECT scans showed that most of the lesions manifested with two enhancement patterns: “fast in and fast out” and “fast in and slow out,” with the former being predominant. However, on CT imaging, the most common types of liver malignancies, such as hepatocellular carcinoma, predominantly manifest the “fast in and fast out” enhancement pattern, whereas hyperplastic nodules of the liver manifest the “fast in and slow out” enhancement pattern (rapid enhancement in the HAP and slightly high density or iso-density in the PVP and delayed phases) (11). Therefore, it was difficult to distinguish PEComa from other focal liver lesions due to the lack of specific imaging features. In 11 cases, most of them showed inhomogeneous enhancement (9/11) and a few showed large blood vessels (4/11), which were similar to those reported in the literature. It has been reported that the CECT of PEComa often shows inhomogeneous enhancement with large and tortuous vascular shadows (20, 22).

As a new technology, CEUS has played an important role in the diagnosis and treatment of tumors (26, 27). In one study, the detection rate of small HCC using CEUS was suggested to be higher than that of CT and even that of MRI (28). However, few studies have reported on CEUS examinations of PEComa patients. In 11 cases, lesions on CEUS showed rapid whole-lesion enhancement within 20 s and mass-like enhancement in the HAP, while in the PVP and delayed phase, the lesions showed a “slow washout” (washout at approximately 1 min in some cases, washout after 3 min in most patients, and no signs of washout even after 360 s in some patients). Hence, the washout in PEComa lesions is significantly delayed compared with that in other malignant tumors, such as HCC and metastatic liver cancers. This enhancement pattern is more frequently associated with imaging of benign tumors (29–31), such as hyperplastic nodules and focal nodular hyperplasia (FNH). This finding is also consistent with clinical manifestations of the disease: slow growth with a predominantly good prognosis. On pathological examination, most PEComas were composed of epithelioid cells with low mitotic activity, and lacked tumor cell necrosis. In our study, all the lesions displayed rapid and homogeneous mass-like enhancement during the HAP and did not show the non-enhanced area that could indicate necrosis on CEUS. These CEUS findings were consistent with pathological demonstrations.

Therefore, compared with CT and CECT, ultrasonography can provide more imaging features, which may be beneficial to the diagnosis and treatment of PEComa. For example, some liver lesions, such as hyperplastic nodules, often do not have an abundant blood supply, and few are surrounded by large blood vessels. These features are markedly different from those of PEComa. FNH has special imaging features, such as central scarring (30, 31). These lesions are easily distinguishable from those of PEComa. Thus, if the lesion shows distinctive ultrasound imaging features, including large blood vessels around the lesion, peripheral hyperechogenicity, the lateral shadow, abundant blood supply to the lesion, and the “fast in and slow out” enhancement pattern, it is highly likely to be PEComa. It is evident that ultrasound imaging tests are slightly superior to CT examinations for the diagnosis of PEComa.

In summary, we retrospectively analyzed the imaging features of PEComas in 11 patients using two types of imaging tests: CT/CECT and US/CDFI/CEUS. The lesions were not found to display any apparent specific characteristics on CT or CECT, indicating that these tests were of little help in the diagnosis of the disease. However, ultrasound imaging presented clear and specific imaging manifestations of the lesions in the patients, which were helpful in the diagnosis of PEComa. Evidently, more patients with PEComa are still needed to further explore and identify relevant imaging features of the disease.

## Data availability statement

The original contributions presented in the study are included in the article/supplementary material. Further inquiries can be directed to the corresponding authors.

## Ethics statement

The studies involving human participants were reviewed and approved by the Ethics Committee of Peking University Cancer Hospital & Institute. The patients/participants provided their written informed consent to participate in this study. Written informed consent was obtained from the individual(s) for the publication of any potentially identifiable images or data included in this article.

## Author contributions

XG and WW performed drafted the manuscript and the study design. XG, WW, HT, QY, JW, HW, YW, MM, KY, and WY performed the imaging studies of the patients and the data analysis. XG, HT, HW, and WW take responsibility for the data collection and sorting. All authors contributed to the article and approved the submitted version.

## Funding

This study was received funding from the Capital Health Research and Development (Grant Number 2020-2-2152), the Beijing Science and Technology Planning Project (Grant Number Z131107002213147), and the Natural Science Foundation of Beijing (Grant Number 7222172).

## Acknowledgments

We thank Prof. Minhua Chen for her help in the research.

## Conflict of interest

The authors declare that the research was conducted in the absence of any commercial or financial relationships that could be construed as a potential conflict of interest.

## Publisher’s note

All claims expressed in this article are solely those of the authors and do not necessarily represent those of their affiliated organizations, or those of the publisher, the editors and the reviewers. Any product that may be evaluated in this article, or claim that may be made by its manufacturer, is not guaranteed or endorsed by the publisher.

## Abbreviations

PEComa, perivascular epithelioid cell tumor; CECT, contrast-enhanced computed tomography; CEUS, contrast-enhanced ultrasound; FLL, focal liver lesion; HCC, Primary hepatocellular carcinoma; MRI, magnetic resonance imaging; CDFI, color Doppler flow imaging; HAP, hepatic arterial phase; PVP, portal venous phase; AFP, alpha-fetoprotein; CEA, carcinoembryonic antigen; CA19-9, carbohydrate antigen 19-9; CA125, cancer antigen 125; HMB45, human melanoma black-45; FIFO, fast in and fast out; FISO fast in and slow out; SMA, smooth muscle actin; AML, angiomyolipoma; CCST, clear cell “sugar” tumor of lung; LAM, lymphangioleiomyomatosis; CCMMT, clear cell myomelanocytic tumor; FNH, focal nodular hyperplasia.
